# Liver transcriptomics reveals features of the host response in a mouse model of dengue virus infection

**DOI:** 10.3389/fimmu.2022.892469

**Published:** 2022-08-26

**Authors:** Wenjiang Zheng, Qian Yan, Zonghui Li, Xianyang Wang, Peng Wu, Feng Liao, Zizhao Lao, Yong Jiang, Xiaohong Liu, Shaofeng Zhan, Geng Li

**Affiliations:** ^1^ The First Clinical Medical School, Guangzhou University of Chinese Medicine, Guangzhou, China; ^2^ The First Affiliated Hospital, Guangzhou University of Chinese Medicine, Guangzhou, China; ^3^ Animal Experiment Center, Guangzhou University of Chinese Medicine, Guangzhou, China; ^4^ Shenzhen Hospital of Integrated Traditional Chinese and Western Medicine, Shenzhen, China

**Keywords:** dengue virus, mouse model, liver injury, transcriptomics, inflammation, host response

## Abstract

**Background:**

Dengue virus (DENV) infection induces various clinical manifestations and even causes organ injuries, leading to severe dengue haemorrhagic fever and dengue shock syndrome. Hepatic dysfunction was identified as a risk predictor of progression to severe disease during the febrile phase of dengue. However, the underlying mechanisms of hepatic injury remain unclear.

**Methods:**

A model of dengue disease was established in *IFNAR* ^−/−^ C57BL/6 mice by challenge with DENV-2. Body weight, symptoms, haematological parameters and liver pathological observations in mice were used to determine the effects of DENV infection. Liver transcriptome sequencing was performed to evaluate the features of the host response in *IFNAR* ^−/−^ mice challenged with DENV. Functional enrichment analysis and analysis of significantly differentially expressed genes (DEGs) were used to determine the critical molecular mechanism of hepatic injury.

**Results:**

We observed haemoconcentration, leukopenia and liver pathologies in mice, consistent with findings in clinical dengue patients. Some differences in gene expression and biological processes were identified in this study. Transcriptional patterns in the liver indicated that antiviral responses to DENV and tissue damage *via* abnormal expression of proinflammatory cytokines were induced. Further analysis showed that the upregulated DEGs were significantly enriched in the leukocyte transendothelial migration, complement and coagulation cascades, and cytokine-cytokine receptor interactions signalling pathways, which are considered to be closely associated with the pathogenic mechanism of dengue. IL6, IL 10, ICAM-1, VCAM-1, MMP9 and NLRP3 were identified as biomarkers of progression to severe disease.

**Conclusions:**

The interactions of these cytokines, which activate inflammatory signalling, may lead to organ injury and haemoconcentration and even to vascular leakage in tissues, including the mouse liver. Our study identifies candidate host targets that could be used for further functional verification.

## 1. Introduction

Dengue, an acute disease caused by infection with an acute arthropod-borne virus and the most common arboviral disease worldwide, imposes a large health and socioeconomic burden on many tropical and subtropical regions (1). There are four serotypes of the dengue virus (DENV) namely DENV-1, DENV-2, DENV-3, and DENV-4. A fifth serotype (DENV-5) has been detected in Sarawak state of Malaysia in October 2013 (2). Although most dengue virus infections are asymptomatic, varied clinical manifestations may occur, ranging from mild febrile illness to severe and fatal disease, inducing dengue haemorrhagic fever/dengue shock syndrome (DHF/DSS) (3). Severe dengue is a potentially deadly complication due to plasma leaking, fluid accumulation, respiratory distress, severe bleeding, or organ impairment and proper medical care is needed to avoid complications and the risk of death (4). The search for biomarkers that reliably predict progression to severe dengue in symptomatic patients is becoming a principal focus of current research efforts.

A systematic review of 122 clinical studies identified that hepatic dysfunction is a risk predictor of the development of severe disease during the febrile phase of dengue (5). DENV targets the liver during the course of the disease, in advanced forms autopsies revealed signs of liver damage, such as mononuclear cell infiltration and mitochondrial swelling. DENV antigen was found in hepatocytes and surrounding the necrotic foci (6, 7). Postmortem studies revealed deformed liver architecture and red blood cell extravasation with the collapse of the reticular formation in sever dengue patients (8). In mouse models, increased number systemic levels of dengue virus particles were found in liver endothelial cells (9). Liver involvement was found to consistently occur in severe dengue both in humans and mice, and the liver is the most commonly affected organ in fatal dengue cases. Although hepatic dysfunction is a hallmark of severe dengue, the role of liver injury in the pathogenesis of dengue virus infection has not been elucidated, especially the core host factors during the liver injury.

Impairment of microvascular function and endothelial dysfunction were associated with severe disease progression (10). Mechanistically, dengue-related liver injury can be both virus-induced and immune-mediated. Liver involvement has been reported in association with cytopathic effects of the virus in dengue patients (11). Dengue NS1 protein acts *via* TLR4 on peripheral blood mononuclear cells, inducing the production of pro-inflammatory cytokines and increasing endothelial permeability (12, 13). Additionally, human MMP-9 protein concentrations rise with DENV NS1 production, further animal studies showed that DENV-2 NS1 activated the enzymatic activity of MMP-9 and synergistically induce vascular leakage (14). In dengue mouse models, early infiltration of natural killer cells, T cells into the liver, and apoptosis of hepatocytes have been shown to be both direct viral infection-induced (15). Earlier research demonstrated that DENV activates the NLRP3 inflammasome and releases IL-1 to participate in platelet shedding, finally resulting in plasma leakage (16). Therefore, the possible mechanisms of dengue-induced liver injury are a combination of direct viral injury to hepatocytes, dysregulation of immune responses to dengue infection and ischemic liver injury from capillary leakage syndrome (17).

Indeed, there may be some potential confounders of hepatic dysfunction in dengue patients, leading to the non-specific elevation of aminotransferases, such as co-infection with chronic hepatitis viruses. However, exclusion of patients positive for hepatitis B or C still showed strong and early aminotransferase increases within days of initial dengue infection signs (18). Therefore, although the role of liver dysfunction in the progression to severe disease is controversial, while a large number of studies show that there is a significant association between hepatic dysfunction and severe dengue patients, it is still necessary to explore the underlying mechanism of liver involvement in dengue.

Recent studies in the mouse model suggested that targeting the host response is a promising therapeutic strategy to improve liver injury. JNK1/2, MAPK and ERK1/2 inhibitor treatment can reduce DENV-induced liver injury (19–21). RNA-Seq study provides a transcriptional map of immune activation in DENV target organs and the edema with dilated liver sinusoids and mononuclear cell infiltration was observed in immune-competent mice infected with the four different DENV serotypes (22). Thus, some host signallings and therapeutic targets had been explored in these non-lethal mouse models. However, the global features of the host inflammatory and immune responses in the liver in a mouse model of dengue virus infection remain unclear, especially the transcriptional response in liver injury, which may reveal the potential therapeutic targets from an overall perspective.

Our previous studies found that DENV activates IL-1β to induce liver tissue injury and vascular leakage in mice, which recapitulated the pathological changes that have been described in human studies (14, 23, 24). In this study, we aimed to evaluate the transcriptomics features of liver injury in a mouse model of dengue virus infection based on a lethal animal model, and then perform a series of functional analyses to partially elucidate the underlying pathogenesis of DENV-induced liver injury.

## 2. Materials and methods

### 2.1 Ethics statements and facility

All methodology involving mice and the experimental protocols in this study were approved by the Animal Ethics Committee of Guangzhou University of Chinese Medicine. Animals were handled in accordance with the Animal Ethics Procedures and Guidelines of the People’s Republic of China and the principles described in the Animal Welfare Act. Experiments involving dengue virus were performed in the Animal Biological Safety Level-2 (ABSL-2) laboratory of Guangzhou University of Chinese Medicine (Guangzhou, China). We have received special training in pathogen management, and we were instructed by qualified viral scientists (especially scientists from the State Key Laboratory of Virology or the Wuhan Institute of Virology of the Chinese Academy of Sciences).

### 2.2 Cell culture

An *Aedes albopictus* gut cell line (C6/36) was purchased from the American Type Culture Collection (ATCC). C6/36 cells were cultured in minimal essential medium (MEM) supplemented with 10% foetal calf serum, 100 U/ml penicillin, and 100 μg/ml streptomycin and maintained at 30°C in a 5% CO_2_ incubator.

### 2.3 Virus amplification

The TSV01 strain of DENV2 (GenBank accession number: AY037116.1, available at https://www.ncbi.nlm.nih.gov/nuccore/AY037116.1) was kindly provided by Dr Wenxin Li of the College of Life Sciences, Wuhan University, China. To generate dengue virus for further animal experiments. C6/36 cells were incubated with DENV-2 at an MOI of 0.5 for 2 h, and unbound dengue virus was then removed by washing. The infected C6/36 cells were then grown in fresh medium supplemented with 2% FBS for seven to ten days. To wash away cellular debris, we harvested the supernatants and centrifuged them at 4000 rpm for 10 min. Then, we used a filter membrane (0.22 μm) to collect dengue virus. All dengue virus was aliquoted into tubes for freezing at -80°C and stored in an ultra-low temperature freezer (Thermo Scientific) in the Animal Laboratory Center of Guangzhou University of Chinese Medicine (Guangzhou, China).

### 2.4 DENV-2 infection models in mice

*IFNAR^−/−^* C57BL/6J mice (deficient in both the IFNα and IFNβ receptors) were provided as gifts by Professor Zhao Jincun of the State Key Laboratory of Respiratory Diseases of Guangzhou Medical University, China. *IFNAR^-/-^* mice on the C57BL/6J background were generated and bred in-house under specific pathogen-free conditions in our laboratory at Guangzhou University of Chinese Medicine. Mice were housed at 21°C in 55% humidity on a 12:12 h light: dark cycle. They were provided access to food and water ad libitum. Ten mice were randomly allocated to the control and experimental groups. Male mice (6-8 weeks old) were challenged intraperitoneally (i.p.) with the DENV-2 TSV01 strain (dengue virus infection group, *n*=5, 1×10^5^ PFU) within an animal biological safety cabinet (ABSL II). Mice injected with an equivalent volume of PBS (mock infection) were used as negative controls (*n*=5). Mice were observed daily for body weight loss and the development of virus-induced disease. At 3, 5 and 8 dpi, blood was taken from the fundus venous plexus to determine viremia and the viral load in the blood. On Day 8 after infection, mice were euthanized with pentobarbital, and the livers were collected and stored at -80°C until further use. Tissues of mice in the two groups were collected for histopathological analysis. When a humane end point (body weight loss of ≥20%, hunched posture, ruffled fur, conjunctivitis, movement impairment, lower limb paralysis) was reached, mice were euthanized.

### 2.5 Mice viral RNA isolation and qRT-PCR

The viral RNA was isolated using a standard protocol (Ultrapure RNA Kit, CWBIO, Guangzhou, China), the RNAs (1 μg) were then reverse transcribed to cDNA by HiScript II Reverse Transcriptase (Vazyme Biotech Co., Ltd. Nanjing, China). The cDNA then was used as templates for RT-qPCR assays using SYBR Green according to the manufacturer’s protocol (BIO-RAD, California, USA). It was performed by the following procedure: heat activate polymerase at 95°C for 10 min, afterward, 45 cycles of 95°C for 15s, 60°C for 15s and 72°C for the 20s, the fluorescence was collected and analyzed at the 72°C steps. A final melting curve step from 55°C to 95°C was used to test the specificity of the primer (23). The absolute copy numbers of animal samples were derived from the Ct values, by reference to a standard curve. Ct values for the known concentrations of RNA were plotted against the logs of the genome equivalent copy numbers. The resultant standard curve was used to determine the number of dengue RNA genome equivalents in the samples. Viral RNA quantification was conducted as previously described (25). The primers used in RT-qPCR detection were listed: DENV-2 forward 5’-CATTCCAAGTGAGAATCTCTTTGTCA-3’, reverse 5’-CAGATCTCTGATGAATAACCAACG-3’.

### 2.6 Haematology

For whole blood analysis, blood was collected from mice *via* the submandibular vein using a 1 ml syringe needle. Blood (500 µl) was collected in 1 ml EDTA-K2 tubes to prevent clotting and was then briefly vortexed, and whole blood analysis was carried out using an automatic animal blood cell analyser (Mai Rui, China, BC-2800vet). The haematological parameters analysed included the red and white blood cell counts, haemoglobin concentration, haematocrit, and platelet count.

### 2.7 Histology

For histopathological staining, mouse liver tissue was fixed in a 5 mL EP tube filled with 4% paraformaldehyde solution for 48 h. Sections were stained with haematoxylin and eosin (HE) for visualization of cell infiltration, oedema, and haemorrhage in the tissues by microscopy.

### 2.8 RNA isolation from mouse liver tissue and library preparation

Total RNA was extracted from mouse liver tissue using TRIzol reagent according to the manufacturer’s protocol (mirVana™ miRNA ISOlation Kit, Ambion-1561). RNA purity and quantity were evaluated using a NanoDrop 2000 spectrophotometer (Thermo Scientific, USA). RNA integrity was assessed using an Agilent 2100 Bioanalyzer (Agilent Technologies, Santa Clara, CA, USA). Then, libraries were constructed using a TruSeq Stranded mRNA LT Sample Prep Kit (Illumina, San Diego, CA, USA) according to the manufacturer’s instructions.

### 2.9 RNA sequencing

Libraries were sequenced on the Illumina HiSeq X Ten platform, and 150 bp paired-end reads were generated. Raw reads in fastq format were first processed using Trimmomatic (26), and the low-quality reads were removed to obtain the clean reads. Then, the clean reads for each sample were retained for subsequent analyses. The clean reads were mapped to the *Mus musculus* genome (GRCm39) using HISAT2 (27). The fragments per kb of transcript per million mapped reads (FPKM) (28) was calculated using Cufflinks (29), and the read counts of each gene were obtained with HTSeq-count (30).

### 2.10 Identification of differentially expressed genes and functional enrichment

Differential gene expression analysis was performed using the DESeq2 (2014) R package (31). An adj. *P* value < 0.05 and a fold change >2 were set as the thresholds for significant differential expression. Hierarchical cluster analysis of differentially expressed genes (DEGs) was performed to demonstrate the expression pattern of genes in different groups and samples. GO term enrichment (http://www.geneontology.org) (32) and KEGG (http://www.genome.jp/kegg) (33) pathway enrichment analyses of DEGs were performed using R based on the hypergeometric distribution.

### 2.11 Statistical analysis

Data are expressed as the mean ± SEM values. Comparisons between two groups were performed using an unpaired *t* test. Differences with a probability value of *p* < 0.05 were defined as significant. GraphPad Prism (https://www.graphpad.com/) and R software (https://www.r-project.org/) were used for statistical analyses and visualization.

## 3. Results

### 3.1 DENV-2 challenge in *IFNAR* ^−/−^ C57BL/6 mice

**Figure 1:**
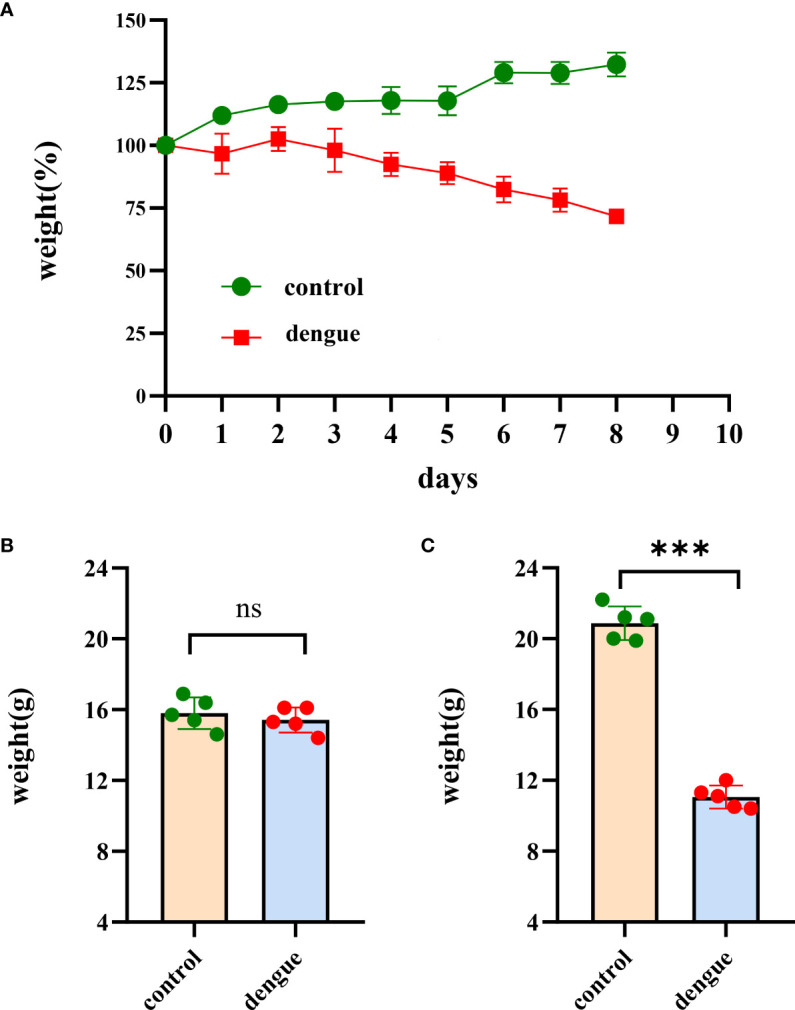
Body weight changes. Changes in body weight in the two groups of mice in the experiments. **(A)** The body weight of mice (n = 5 per group) was monitored and recorded daily after inoculation with DENV-2 (TSV01) until 8 dpi. The weight is expressed as the percentage of the initial weight. While the bodyweight of mock-infected mice gradually increased, DENV2(TSV01)-infected mice lost weight from days 1 to 8 post infection. **(B)** Initial weight in the two groups of mice. **(C)** Body weight of mice at 8 dpi. The data are representative of two independent experiments. Comparisons between two groups were performed using an unpaired *t* test. Each dot represents one mouse, and the data are expressed as the mean ± SEM values for groups of five mice. ns, not significant; ***P ≤ 0.001.

**Figure 2:**
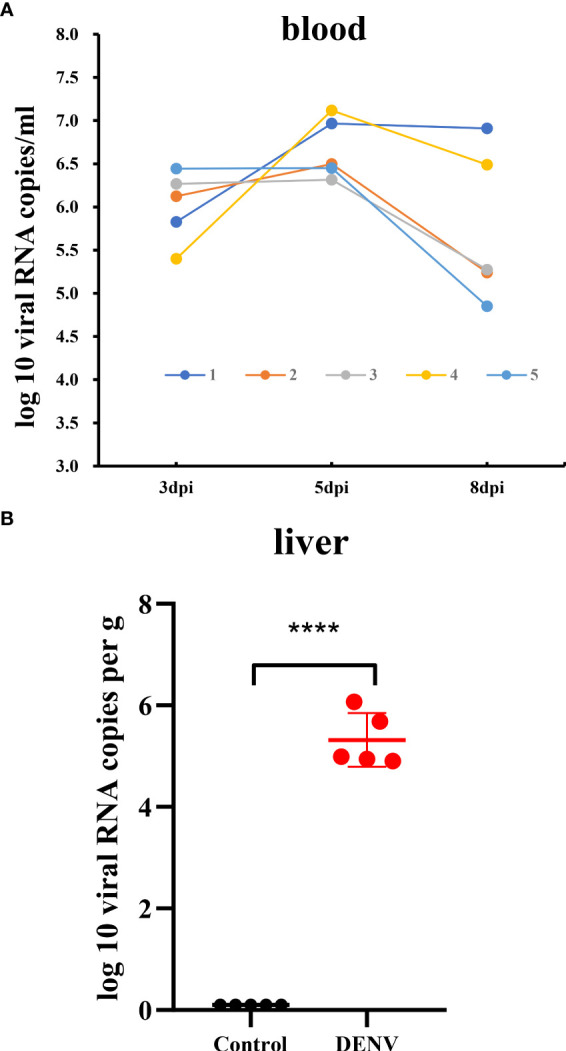
Viral RNA load in the blood and liver tissue of the dengue virus-infected mice. **(A)** Time-course of dengue viral RNA in the sera of the mice collected at 3, 5 and 8dpi. **(B)** Viral RNA load in liver tissue of mice. The viral RNA load in liver of mice was determined by RT-qPCR. Data were shown as mean ± SEM. ****P ≤ 0.0001.

In a preliminary experiment, we demonstrated that DENV successfully infected *IFNAR* ^–/–^C57BL/6 mice deficient in the IFN-α/β receptors. To reveal the features of the inflammatory and immune responses to dengue virus infection in animals, an *IFNAR* ^–/–^ C57BL/6 mouse model was established, and the animals were infected with DENV (TSV01). Control animals were administered culture medium instead of virus. After challenge, mice began to show clinical signs such as weight loss, listlessness, ataxia, and paralysis of one or both hind limbs between 1 and 8 dpi. Mice in the control group did not show any clinical manifestations or signs of weight loss. On Day 0 of virus infection, there was no statistically significant difference in body weight between the two groups of mice; on the 8th day, there was a statistically significant difference **(** **Figure 1** and **Supplementary Table S1**). We then measured the viral load in the blood of dengue virus-infected mice (**Figure 2** **)**.

### 3.2 Haematological parameters in DENV-infected mice

**Figure 3:**
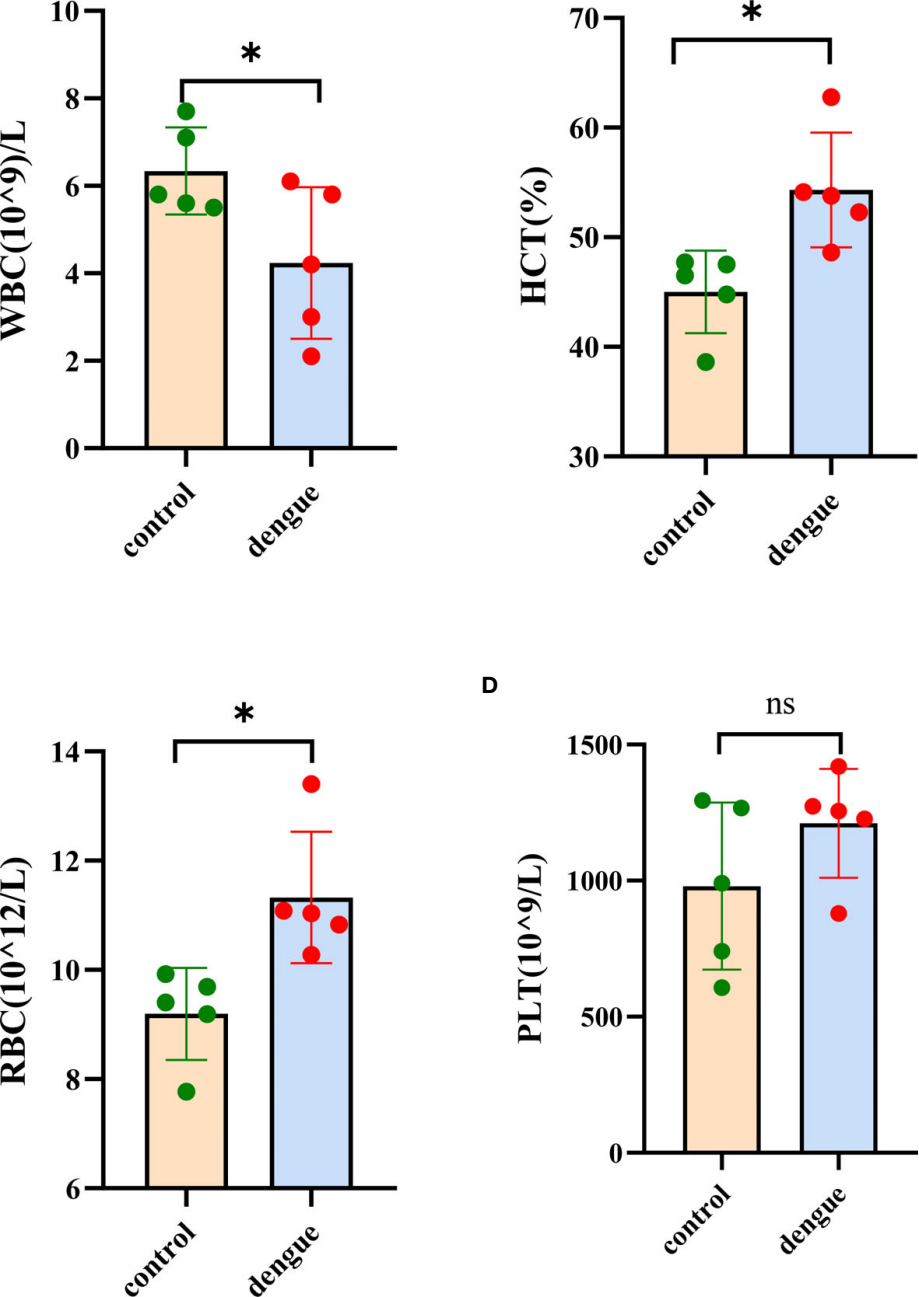
Haematological analysis. Blood samples were collected for haematological analysis eight days post infection. **(A)** White blood cell (WBC) count, **(B)** haematocrit (HCT), **(C)** red blood cell (RBC) count and **(D)** platelet (PLT) count. The results were obtained from two independent experiments groups. Comparisons between two groups were performed using an unpaired *t* test. Each dot represents one mouse, and the data are expressed as the mean ± SEM values for groups of five mice. ns, not significant; *P ≤ 0.05.

Haematological analysis showed that DENV-infected mice exhibited a decreased white blood cell count (WBC) and significant increases in both the red blood cell (RBC) count and haematocrit (HCT) compared to those in uninfected mice **(** **Figures 3A–C** and **Supplementary Table S2** **)**. These results suggest that DENV-infected mice exhibited leukopenia and haemoconcentration. In this experiment, we did not observe thrombocytopenia in DENV-infected mice **(** **Figure 3D** and **Supplementary Table S2** **)**.

### 3.3 Liver pathological observations in DENV-infected mice

**Figure 4:**
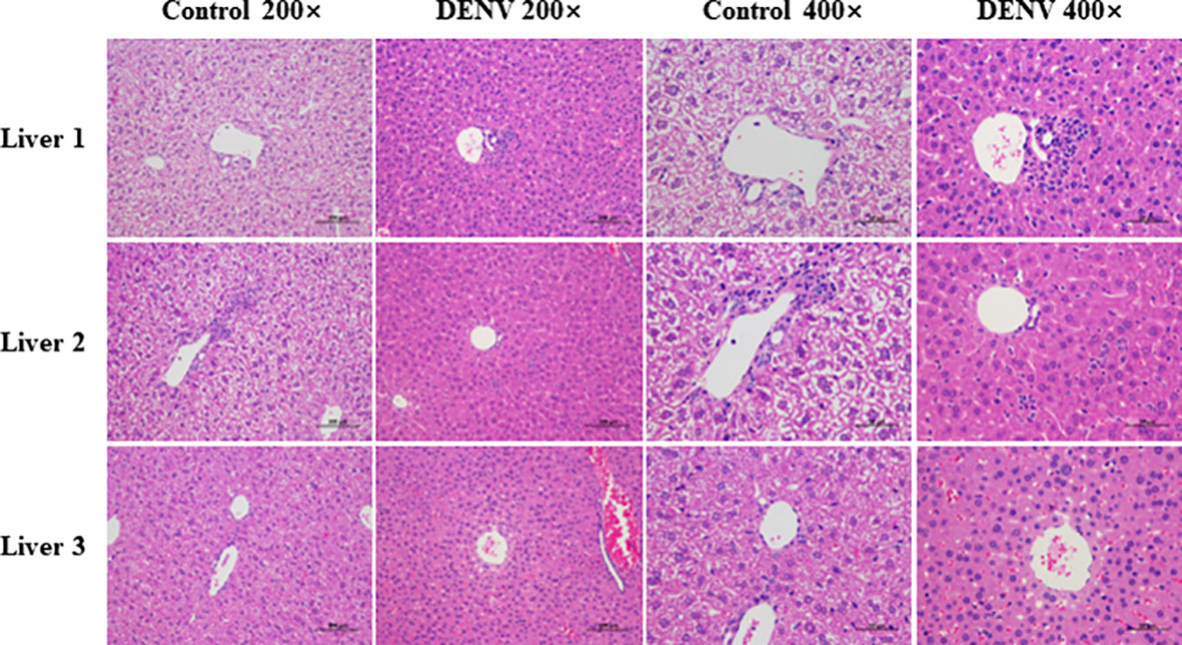
Histopathological analysis. Liver tissues were fixed with 4% paraformaldehyde and subjected to HE staining eight days post infection (magnification: 200× scale bar = 100μm, and 400× scale bar =50 μm). Hepatocytes of mice in the dengue virus infection group were observed to have significant enlargement and translucency. The hepatocyte morphology changed from polygonal to spherical, accompanied by inflammatory cell infiltration. The results shown are representative of 3 individual mice from each group.

Eight days after infection, liver tissues were fixed with 4% paraformaldehyde for histopathological analysis and H&E staining. Histopathological analysis of DENV-infected mice revealed signs of liver injury, including widespread swelling of hepatocytes and focal mononuclear inflammatory cell infiltration, compared to the conditions in uninfected mice. In addition, significant enlargement and translucency of hepatocytes were observed, with hepatocytes showing voluminous cytoplasm and a morphological change from a polygonal to a spherical shape, accompanied by inflammatory cell infiltration (**Figure 4**).

### 3.4 RNA sequencing and gene expression statistics

**Figure 5:**
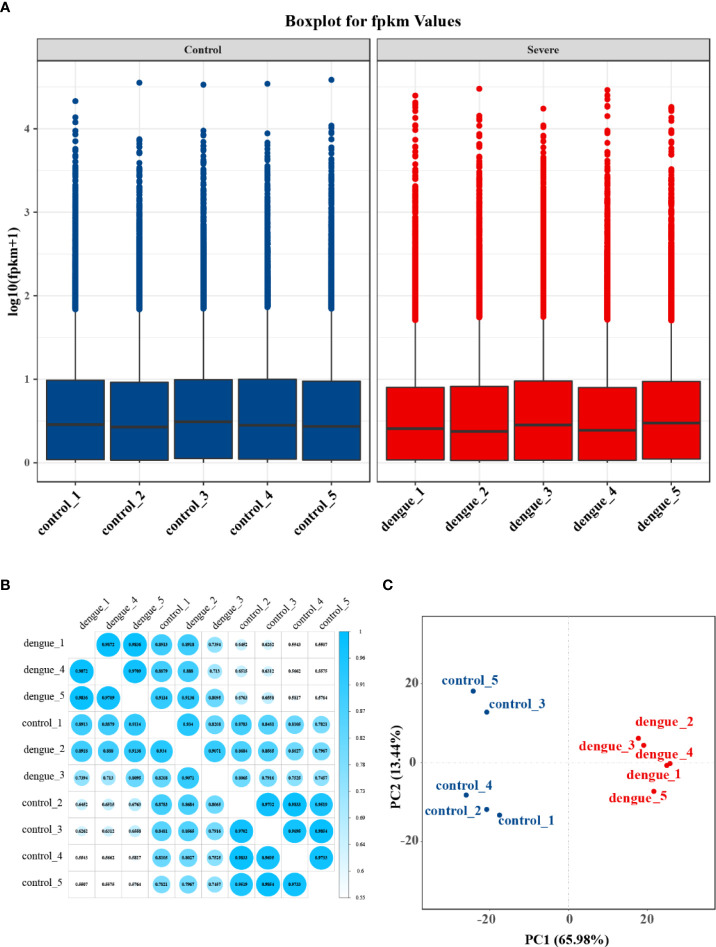
Gene expression statistics of RNA-Seq. **(A)** Boxplot of FPKM values for each independent sample. The box-and-whisker plot shows the dispersion degree of the data distribution. The horizontal axis shows the sample name, the vertical axis shows the log10(FPKM+1) values, and each area of the box plot corresponds to five statistics (top to bottom: maximum, third quartile, median, first quartile and minimum values). **(B)** Plot of correlation coefficients between sequencing samples. The horizontal axis shows the sample name, the vertical axis shows the corresponding sample name, and the colour represents the magnitude of the correlation coefficient. **(C)** Principal component analysis (PCA) was performed using the quantitative results of gene expression to investigate the distribution of samples, and the samples in the same group in this experiment exhibited a more concentrated spatial distribution.

To investigate the differential gene expression between the livers of mice with dengue virus infection and healthy control mice, we compared gene expression at the transcriptional level by RNA-Seq. After excluding low-quality reads, a total of 65.54 GB of clean data were obtained; the platform generated an average of 6.5 GB of data. The high-quality reads of all samples showed a mapping rate of over 93%. The Q30 values of all sequences in the ten cDNA libraries exceeded 93%, and more than 93% of the bases were valid. The FPKM method can eliminate the influence of differences in protein-coding gene lengths and sequencing read quantities on the calculated expression levels of protein-coding genes, and the calculated gene expression levels reflect high or low expression. The gene expression levels in each sample are shown in **Supplementary Table S3**. The statistical table of the number of reads in each sample is shown in **Supplementary Table S4**. The box-and-whisker plot of gene expression showed a concentrated range of values for the five samples in the two groups, with high similarity between samples **(** **Figure 5A** **)**. Plots of the correlation coefficients between sequencing samples were obtained based on gene expression **(** **Figure 5B** **)**. Principal component analysis (PCA) of gene expression revealed an overall similar pattern of response across the samples, and the samples in the same group exhibited a more concentrated spatial distribution **(** **Figure 5C** **)**. These results indicated that the transcripts of the ten liver samples were all of high quality and could be used for subsequent analysis.

### 3.5 Differentially expressed genes during DENV-2 infection

**Figure 6:**
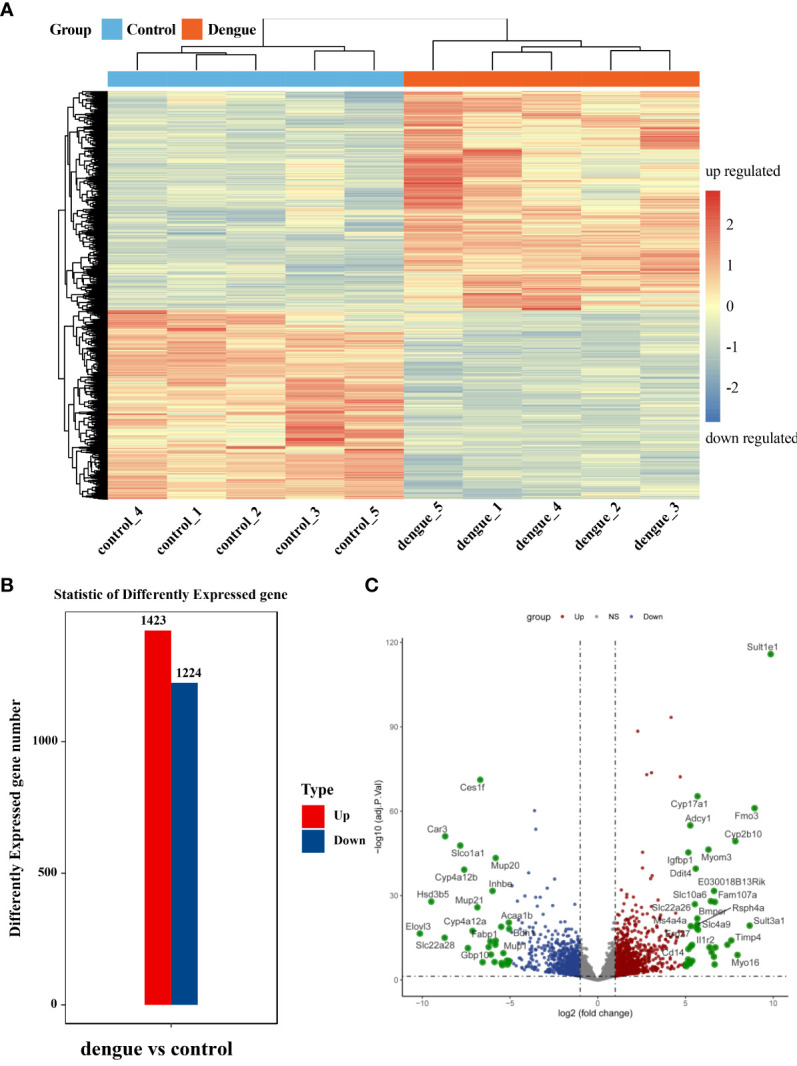
Identification of differentially expressed genes. **(A)** Heatmap of DEGs. Unsupervised hierarchical clustering of differentially expressed genes. Samples from the two independent groups were assigned to the same cluster through cluster analysis. **(B)** Statistical bar graph of differentially expressed genes. **(C)** Volcano plot of all genes in the samples from the two groups. The red dots correspond to upregulated genes, the green dots correspond to downregulated genes, and the grey dots correspond to genes without statistically significant differences in expression. ns, not significant.

Differentially expressed transcripts were screened based on the criteria of log2(fold change) ≥ | ± 1| and adj. *p* value < 0.05. We further screened the top significantly upregulated and significantly downregulated genes in the two groups with a heatmap **(** **Figure 6A** and **Supplementary Table S5** **)**. In total, 2647 genes were significantly differentially expressed in the liver in dengue virus infection model mice: 1423 upregulated and 1224 downregulated genes **(** **Figure 6B** **)**. The characteristics of the differentially expressed genes between the two samples were determined by volcano plots **(** **Figure 6C** **)**.

### 3.6 Functional annotation of transcriptional changes

**Figure 7:**
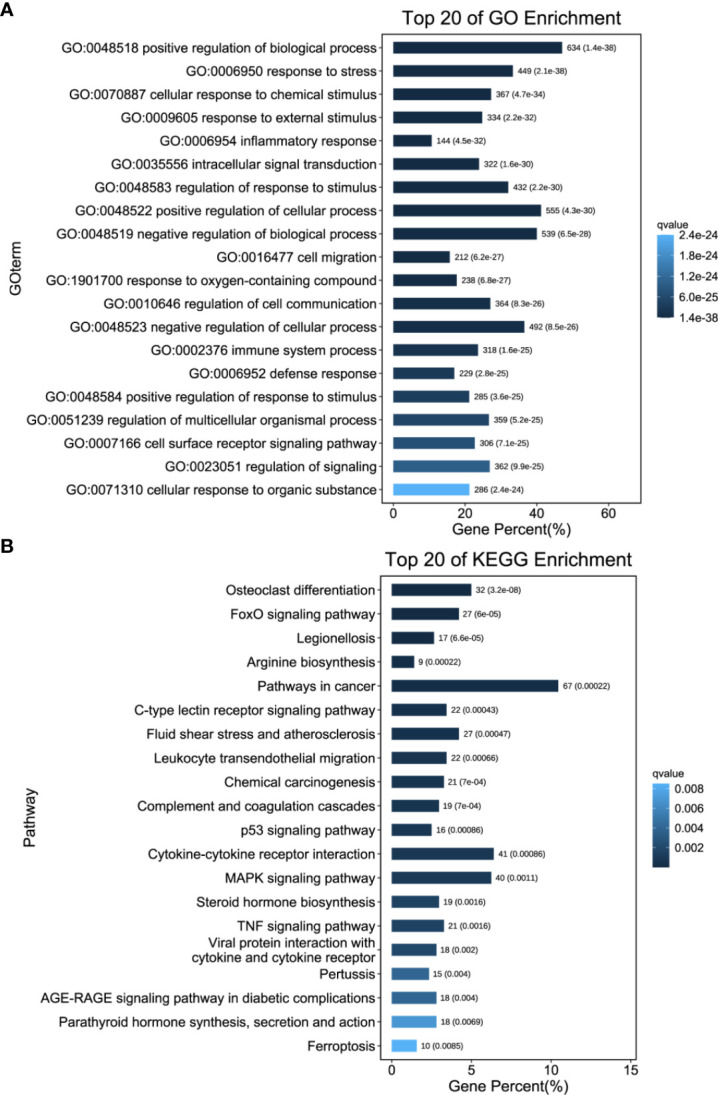
Functional Annotation of Transcriptional Changes. **(A)** GO enrichment analysis was performed on the DEGs to describe their functions. **(B)** KEGG enrichment analysis. The number of DEGs included in each GO or KEGG term was determined, and the significance of DEG enrichment in each term was calculated using the hypergeometric distribution. A lower enrichment *P* value indicates greater statistical significance.

**Figure 8:**
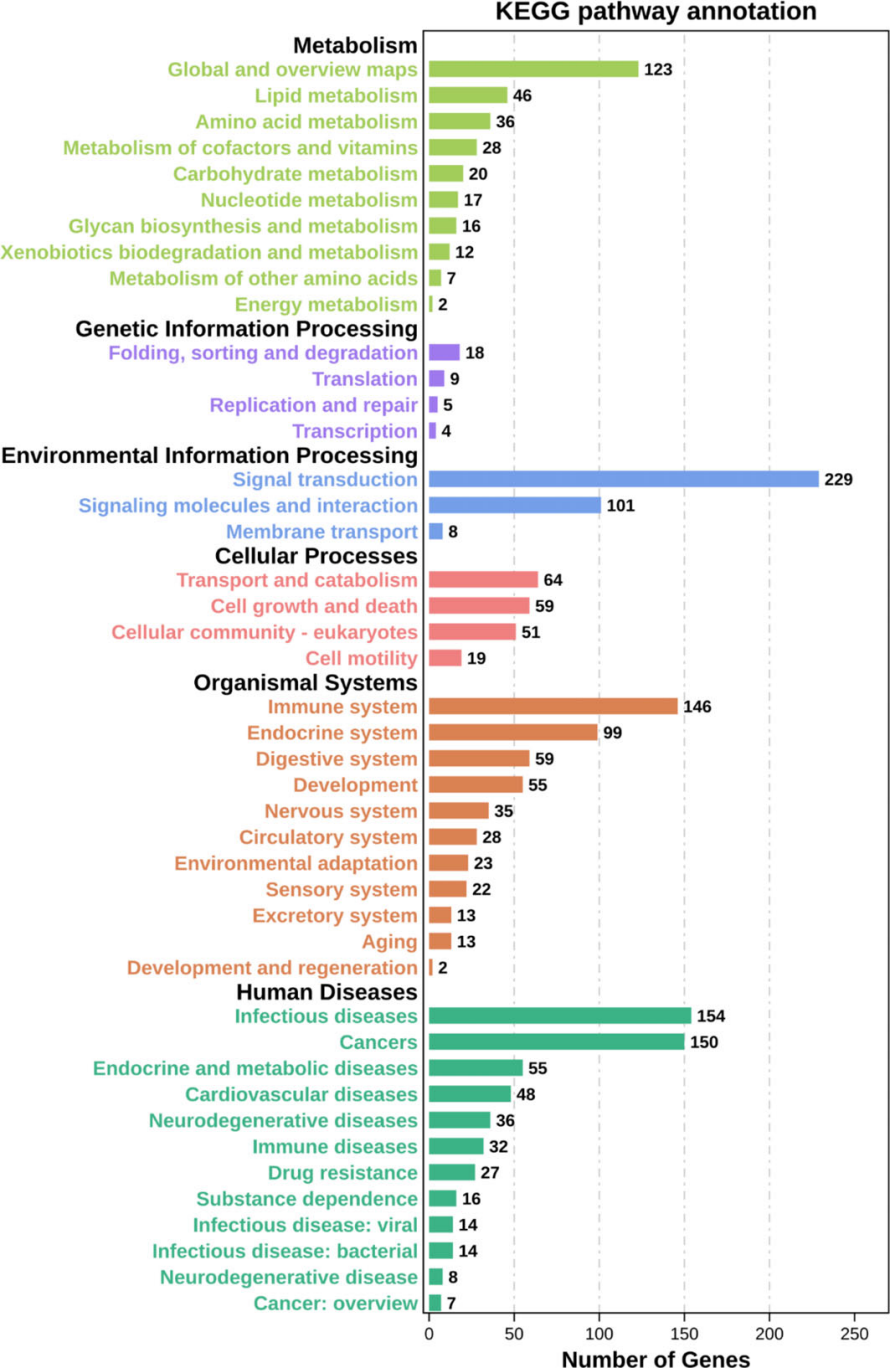
KEGG pathway annotation. Classification statistics of each KEGG pathway with aspects such as metabolism, genetic information processing, environmental information processing, cellular processes, organismal systems, and human diseases.

We then performed GO and KEGG enrichment analyses of these significantly upregulated DEGs to compare the host inflammatory and immune responses in the liver. In the “Biological Process” category of gene ontology, differential expression of genes involved in the inflammatory response, intracellular signal transduction, cell migration, immune system process and defence response was found compared to that in control mouse livers **(** **Figure 7A** and **Supplementary Table S6** **)**. However, genes involved in leukocyte transendothelial migration, complement and coagulation cascades, the p53 signalling pathway, cytokine–cytokine receptor interactions, the MAPK signalling pathway, the TNF signalling pathway, and ferroptosis were activated post DENV infection **(** **Figure 7B** and **Supplementary Table S7** **)**. Taken together, these data showed that DENV infection elicited a broad range of gene expression changes, some of which tracked closely with viral replication and others that may be involved in the host inflammatory and immune responses **(** **Figure 8** and **Supplementary Table S8** **)**.

### 3.7 Identification of hub genes in the livers of DENV-infected mice

**Figure 9:**
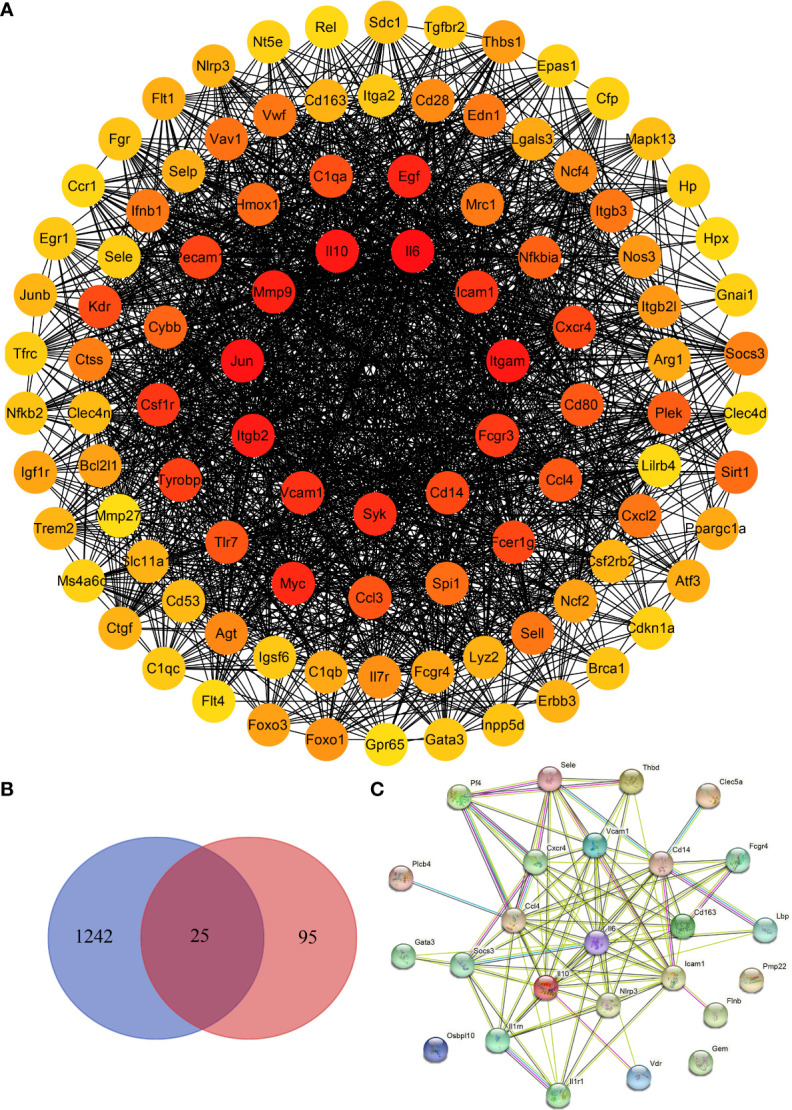
Identification of hub genes in the liver in DENV-infected mice. **(A)** Network topology analysis and protein–protein interaction (PPI) network of the top 100 differentially expressed genes between dengue virus-infected mice and control group mice. **(B)** Venn diagram. **(C)** PPI network of the 25 shared genes.

**Figure 10:**
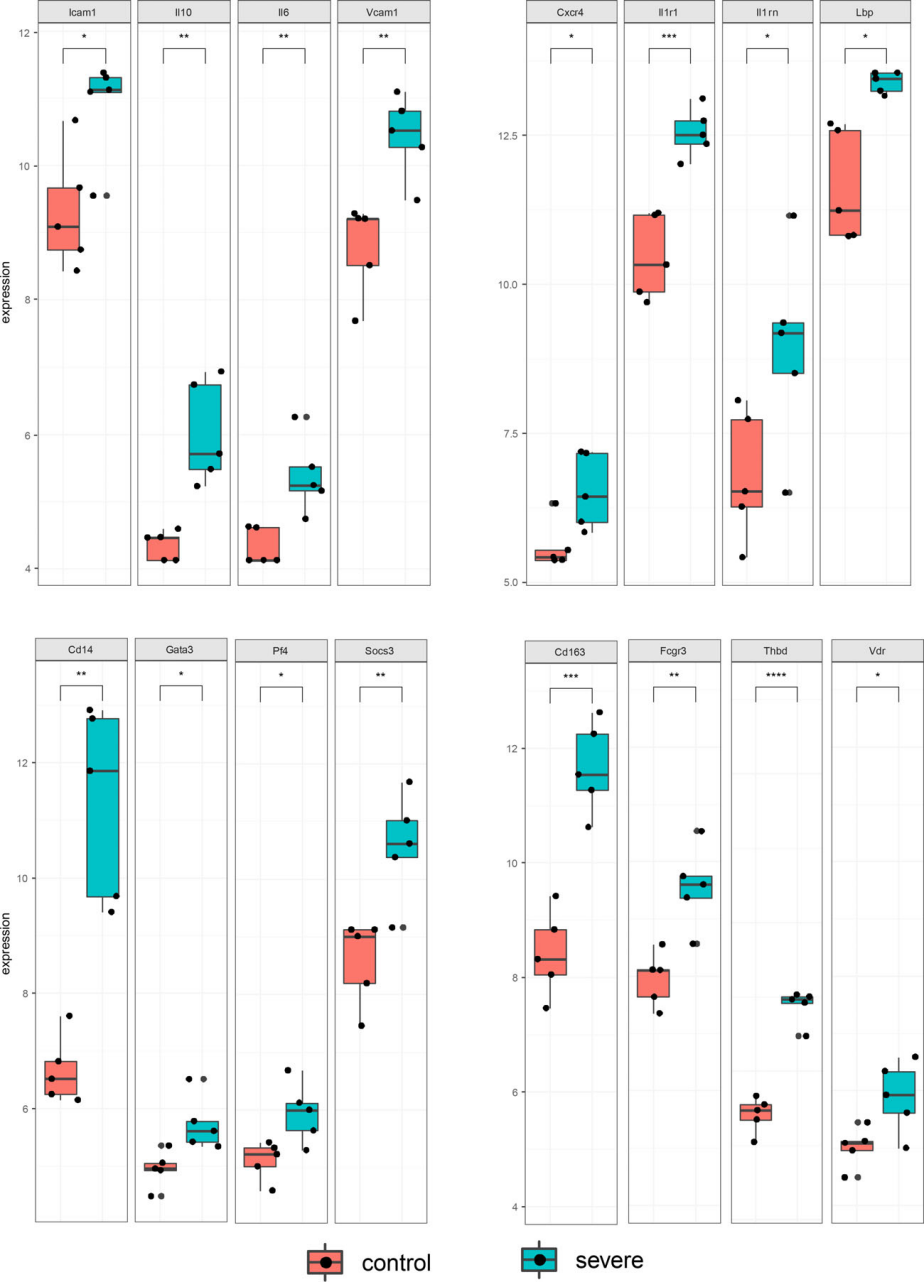
Statistical bar graph of hub genes. The results were obtained from two independent experiments groups. Comparisons between two groups were performed using an unpaired *t* test. Each dot represents one mouse, and the data are expressed as the mean ± SEM values for groups of five mice. ns, not significant; *P ≤ 0.05; **P ≤ 0.01; ***P ≤ 0.001, ****P ≤ 0.0001.

We screened the DisGeNET database to identify 1267 genes associated with severe dengue (**Supplementary Table S9** and **Table S10**). The genes were used for PPI analysis in the STRING database, and the CytoHubba plugin in Cytoscape software was used for network topology analysis and visualization (**Figure 9A** and **Supplementary Table S11**). We then performed tissue enrichment analysis (**Supplementary Table S12**). The Venn diagrams showed 25 shared DEGs between control group mice and mice with dengue virus infection (**Figures 9B, C** **)**. Among those hub genes, ICAM1, IL10, IL6, and VCAM1 had higher degree connectivity values (**Figure 10**).

## 4. Discussion

The current study utilized the *IFNAR* ^–/–^ C57BL/6 mouse model of DENV infection to identify the liver transcriptomic features of the host response in mice with dengue virus infection by RNA-Seq. The results of the experimental study showed that on the fourth to fifth day after the DENV challenge, mice exhibited varying degrees of macroscopically visible clinical signs, including reduced activity and reduced weight gain. The disease condition gradually worsened and continued until the eighth day. We excised liver tissue from DENV-infected mice for pathological observation and found pathological changes, such as inflammatory cell infiltration and oedema in the liver. The whole blood analysis is consistent with observations in humans with dengue disease, both mild and severe, which show similar alterations in haematological features (34). DENV-infected mice experienced a substantial reduction in the white blood cell count and a significant increase in haematocrit in just a few days after DENV infection, which is direct evidence of haemoconcentration (35). By measuring the viral load in the blood of dengue virus-infected mice at 3 different time points, we found that the mice developed a high viremia from 3 dpi to 5 dpi, and there were still two mice experienced a high viral load at 8 dpi, indicating that the mice had experienced a period of dengue virus infection.

Based on transcriptomic analysis, this study also identified some differences in gene expression and biological processes. In particular, the primary host pathways disrupted by DENV2 infection were leukocyte transendothelial migration (TEM), complement and coagulation cascades, cytokine-cytokine receptor interactions, and viral protein interaction with cytokine and cytokine receptors. In addition, intracellular signalling and immune system processes dominated the host response to DENV2 infection. KEGG signalling pathway analysis revealed that MAPK, TNF, FoxO, and ferroptosis are thought to be closely associated with the pathogenesis of dengue. These key pathways may be involved in the progression of DENV infection and partially explain the liver dysfunction seen in dengue patients. Cytokine storm has almost become one of the main accepted mechanisms of severe dengue (36). Programmed cell death may limit pathogen replication in infected cells while at the same time promoting inflammatory and innate responses that generate long-term effective host immunity (37). The complement system is a significant component of the innate immune response (38), and is a double-edged sword that can act as the first line of defence against DENV infection, however, if over-activated, it can also enhance the severity of the disease (39). This finding was corroborated by the complement protein depletion, low C3 levels and serum FH levels observed in patients in the acute phase of DHF (40). Transcriptome analysis in liver tissue showed that DENV infection induces a broad range of host gene responses that characterize the control of viral infection. Most of the important DEGs found in liver samples from individual DENV-infected mouse were associated with inflammatory and defensive responses.

These changes in the liver injury of DENV-infected mice which had been found by RNA-Seq in our study were closely related to patients with acute dengue. Our data indicate that leukocyte transendothelial migration played an important role in the challenge of DENV infection which has few been reported in the previous relevant studies. Leukocyte transmigration was an important process in the inflammatory response, damage or pathogen-associated molecular patterns could induce the release of pro-inflammatory stimulus, which promotes the endothelium to an inflammatory state, and drives the TEM of leukocytes. Although relevant studies were limited, it could be indicated that leukocyte migration would be triggered and driven by DENV infection, based on the mechanism by which it occurred. These responses to DENV infection assisted in infection resolution and played critical roles in protecting against reinfection. However, they might enhance the disease severity, including giving rise to cytokine storm, complement overactivation, coagulation abnormalities, and vascular permeability increase (41).

Among the hub genes, IL10, SOCS3, IL1RN, IL6, VCAM1, IL1R1, CCL4, GATA3, LBP, ICAM1 and PF4 were principally related to cytokine-mediated signalling pathways. IL6, VCAM1 and ICAM1 act as markers of endothelial activation and are involved in the local acute inflammatory response (42). Increased expression of ICAM-1 and VCAM-1 is an *in-situ* indicator of increased vascular endothelial permeability and a subsequent influx of cells that promote endothelial inflammation (43). Previous data from our laboratory showed that the DENV M protein can induce vascular leakage in mouse liver tissue through activation of the NLRP3 inflammasome and IL-1β (23). We identified NLRP3 as a characteristic differentially expressed gene in the current PPI analysis. We suggest that interfering with NLRP3 inflammasome activation may be a feasible strategy to treat DENV-induced endothelial dysfunction (44). IL-6 directly affects endothelial cell permeability by inducing leukocyte recruitment, local inflammation and damage to endothelial cells, causing them to produce several different types of cytokines and chemokines and activating the coagulation cascade (45). Elevated plasma IL-10 can potentially predict the severity of disease in patients with DENV infection to some extent (46–48). Previous studies shown that abnormal inflammasome and monocyte activation play a pathogenic role in the development of dengue, supported by evidence based on clinical samples in which IL-18, LBP and sCD14 are elevated in patients with severe dengue (49). A study of virus-inclusive scRNA-Seq revealed that CD163 was expressed in monocytes only in subjects who subsequently developed severe dengue (50).

These results suggest that microvascular and endothelial dysfunction is associated with dengue virus infection both in mice and patients. We suggest that the inflammatory and host response caused by DENV infection could activate the vascular endothelium, altering permeability and providing a favorable condition for vascular leakage, inducing the occurrence of severe dengue patients. These findings were consistent with the observation *in vitro* experiments, the disorganization of the actin cytoskeleton in DENV-infected cells, reduction of vascular endothelial calcium mucin and platelet endothelial cell adhesion molecule-1 (51). And these changes were associated with the clinical manifestations of petechiae, haemorrhage, decreased blood pressure and hemorrhagic shock in patients with severe dengue; and corresponding with the test results of leucopenia and reduced plasma fluid, was linked to the autopsy pathological findings of perivascular edema and loss of endothelial junctional integrity (52).

**Limitations:** No extensive haemorrhagic necrosis of the liver parenchyma was observed in our study. Most of the histopathological changes reported are based on samples obtained from fatal cases (autopsy stage) which has led to histopathological changes observed in animal experiments that are inconsistent with human samples (8). Indeed, IFNAR-deficient mice are unlikely to reflect the immune response of immunocompetent mice, or to accurately mirror those in humans (53). IFNAR^–/–^ mice provide however a valuable model for supporting the replication and proliferation of DENV, resulting in obvious viremia (54). The unpublished data in our lab suggested that the animal model reproduced a decrease in leukocytes, an increase in hematocrit, and a significant decrease in platelets. Lack of thrombocytopenia was probably due to the small sample size. The purpose of this study was to characterize the host’s early response through the liver transcriptome of the dengue virus infection in this mouse model (the 8th day after dengue virus infection). Therefore, this may be the most significant and most relevant stage of differentially expressed genes. We will report the liver function levels (AST/ALT), liver tissue pathology (histology) and transcriptomic signatures of changes at different times (day 1, day 3, day 5, day 7, etc.) in the future studies to analyze their correlation with differentially expressed genes and reveal the different perspectives on disease dynamics.

## 5. Conclusions

Our findings revealed that DENV invasion in mice leads to significant differential expression of a large number of genes in multiple signalling pathways in the host (especially the leukocyte transendothelial migration, complement and coagulation cascades, cytokine-cytokine receptor interactions, and viral protein interaction with cytokine and cytokine receptors). Most of these differentially expressed genes (IL10, SOCS3, IL1RN, IL6, VCAM1, IL1R1, CCL4, GATA3, LBP, ICAM1 and PF4) are associated with the host viral defence as well as the repair of tissue damage and, in particular, can lead to endothelial dysfunction and increased vascular permeability in liver tissue, further causing the development of vascular leakage.

## Data availability statement

The data presented in the study are deposited in the GEO repository (https://www.ncbi.nlm.nih.gov/geo/), accession number GSE210022.

## Ethics statement

The animal study was reviewed and approved by the Animal Ethics Committee of Guangzhou University of Chinese Medicine (20211008006).

## Author contributions

WZ and QY conceived and designed the study. WZ and QY analysed the RNA-Seq data. WZ, ZHL and XW wrote the manuscript. WZ, ZHL, XW, ZZL, and PW participated in the animal experiments. WZ, QY, PW, ZHL, and XW participated in sample collection. YJ, SZ, XHL and GL revised the manuscript. All authors contributed to the article and approved the submitted version.

## Funding

This research was funded by grants from the “Double First-Class” and High-level University Discipline Collaborative Innovation Team Project of Guangzhou University of Chinese Medicine (Grant No. 2021XK16), Sanming Project of Medicine in Shenzhen (Grant No. SZZYSM202106006), and the Technology Research of COVID-19 Treatment and Prevention and Special Project of Traditional Chinese Medicine Application-Research on the platform construction for the prevention and treatment of viral infectious diseases with traditional Chinese medicine (Grant No. 2020KJCX-KTYJ-130).

## Acknowledgments

We thank Dr Jincun Zhao of the First Affiliated Hospital of Guangzhou Medical University, Guangzhou, China, for the gift of *IFNAR^-/-^* C57BL/6 mice deficient in the IFN-α/β receptors and Dr Wenxin Li of the College of Life Sciences, Wuhan University, China, for DENV2 strain TSV01.

## Conflict of interest

The authors declare that the research was conducted in the absence of any commercial or financial relationships that could be construed as a potential conflict of interest.

## Publisher’s Note

All claims expressed in this article are solely those of the authors and do not necessarily represent those of their affiliated organizations, or those of the publisher, the editors and the reviewers. Any product that may be evaluated in this article, or claim that may be made by its manufacturer, is not guaranteed or endorsed by the publisher.
